# Wouldn’t It Be Nice to Not Fall for It Twice? Prior Experience Does Not Abolish the Impact of Expectancy Violations on Attention Capture

**DOI:** 10.3390/vision10020032

**Published:** 2026-05-29

**Authors:** Isabella Fuchs-Leitner, Gernot Horstmann, Ulrich Ansorge

**Affiliations:** 1 Department of Psychiatry—Specialization Addiction Medicine, Kepler University Hospital, 4020 Linz, Austria; 2 Medical Faculty, Johannes Kepler University Linz, 4040 Linz, Austria; 3 Faculty of Psychology, University of Vienna, 1010 Vienna, Austria; ulrich.ansorge@univie.ac.at; 4 Department of Psychology, Bielefeld University, 33615 Bielefeld, Germany; gernot.horstmann@uni-bielefeld.de

**Keywords:** vision, attention, surprise capture, expectancy violation

## Abstract

In the current study, the impact of an unexpected color swap (from target to distractor color and vice versa) on reaction times (RTs) and eye fixations was investigated. Within a visual search task, participants reported the location of a shape-defined target letter above or below a distractor letter. Across time, participants learned the consistent relationships between the visual target features (shape and color), establishing an according expectancy. To that end, fixed colors were first assigned to both a fixed-shape target (e.g., red) and a fixed-shape distractor (e.g., green). Next, this expectancy was violated by an unannounced swapping of target- and distractor-associated colors in the critical trial. In addition, subsequent color swaps estimated the costs of better anticipated color swaps. Eye movements showed longer fixation times on the distractor letter during and immediately after the critical trial compared to trials before the color swap (precritical trials), and, in line with prior research, we also found longer RTs in critical trials compared to consecutive (postcritical) trials without color swaps (Experiment 1). Finally, varying the number of distractors per trial indicated no set-size effect (Experiment 2), but again costs for all expectancy violations were found. In sum, our results indicate that humans tend to fall for expectancy violations more than once, and costs associated with color swaps cannot be avoided entirely.

## 1. Introduction

Past studies have demonstrated that visual attention—the selection of only part of the visual input (against the backdrop of ignoring other parts of the same input)—depends on observers’ expectancies (e.g., [1,2,3]). Observers’ expectancies oftentimes facilitate the selection of expected or expectancy-confirming inputs (e.g., [4,5,6,7,8]). However, the opposite can sometimes be observed too: stimuli violating an observer’s expectancy can also attract attention and the eyes (e.g., [9,10,11,12,13]). In many cases, the reason for the influence of expectancies is that human perception is based on priors (or prior probabilities) of features, events, or objects experienced in the past. These priors are compared to incoming visual input facilitating processing of expected stimuli [14,15,16,17]. At a functional level, prediction errors signal mismatches between expected and observed input and may trigger updating of attentional prioritization.

Although the effects of expectancy violations and prediction errors on attentional selection are well established, considerably less is known about how multiple learned priors are represented and maintained over time in visual search. In particular, previous work has not clarified whether observers concurrently maintain and flexibly reinstate more than one learned target prior within the same task context. For example, Horstmann and Herwig [11] showed that after learning a target’s fixed color, an unexpected color change led to slower search times and altered eye movements—that is, decreased fixation latencies and increased dwell times, with some effects persisting despite the novel target color being consistent.

In two respects, this research is instructive. First, in addition to expectancy violations, other specific factors influence processing efficiency, including intertrial priming (cf. [6,8]) or target salience (e.g., target status as either a color singleton or a color nonsingleton; cf. [18,19]). This is important because expectancy violations entail less priming (i.e., target color in Trial *N* ≠ target color in Trial *N* + 1) than expected trials (i.e., target color in *N* = target color in *N* + 1), and priming and salience can interact (e.g., [20,21]). Second, prior work also suggested that expectancy violations can induce persisting changes in performance (e.g., [22,23]). However, it often remains unclear whether participants also maintain their initial prior (e.g., for a particular target color in visual search) in working memory, thereby enabling efficient processing of subsequent targets regardless of their color, as long as all of these colors have been experienced to be associated with the target.

To address this question, the expectancy violation was repeated in the current study. Critically, the present study was not designed merely to demonstrate prediction-error effects or attentional capture by expectancy violations again, but rather to test the representational status of learned target priors after repeated and extended learning. We tested whether participants can maintain and use two equally well-learned priors about target color by changing target colors repeatedly. Past research has estimated that priors for target-related expectancies in visual search are based on about the last 50 trials in an experiment [24]. However, this research was conducted with intermixed targets of different colors. In this situation, statistical learning would likely correspond to updates of a single prior (cf. [3]). Thus, previous paradigms cannot determine whether observers retain multiple independent priors or merely continuously update one currently dominant prior representation. Here, we went one step further.

We ensured conclusive acquisition of two target-color associations by extended periods of consistent target coloring for more than 50 trials in a row before a target-color swap occurred. This design allowed us to isolate whether participants can reactivate a previously established target prior after learning an alternative one, rather than simply benefiting from short-term intertrial priming or recent trial history. In this situation, three different results were possible. First, in case two priors have been established, a first prior for Target Color A and a second prior for Target Color B that participants have both conclusively acquired through experience in at least 50 consecutive search trials of the same target color, switching back from Prior B to Prior A could incur no search-time costs. (The same prediction follows if participants can strategically abstain from the incorporation of color into their target-related representations following the first target-color swap. This was tested in Experiment 2.) Second, in this situation of extended periods of acquisition and application of one of two priors, A or B, participants may represent both priors, A and B, but as mutually exclusive alternatives (i.e., Prior A = ⌐Prior B and vice versa). If participants maintain both priors but the priors are represented as mutually exclusive, swapping back from Prior B to Prior A (i.e., after the preceding acquisition of both priors) could incur a larger cost than the initial swap from Prior A to the hitherto unknown Target Color B (i.e., after the preceding acquisition of Prior A only). Third, if only a single prior is updated, as was the case in experiments with intermixed target colors in past research, processing costs may also be relatively equal for initial swaps and later swapping back because no priors for the first target-color association would be maintained.

To be precise, in the present study, we studied attention during an initial expectancy violation and during a second (and sometimes third) expectancy violation. The latter reinstated a previously learned target-color–shape association. In Experiment 1, each search display contained only a target and a distractor (see [12]). In the expectancy-violating (i.e., critical) trials, the target and distractor swapped their colors relative to the learning (i.e., precritical) trials. Such swaps allow testing whether previously acquired target-shape–color associations influence attention during and after the swap (e.g., [6,20,25]).

To minimize potential salience confounds, we used simple two-item displays (with one target and one distractor), thereby reducing, if not eliminating, salience differences between conditions [12]. In addition, we trained two complementary target-shape–color associations (target = Color A/distractor = Color B vs. target = Color B/distractor = Color A), with equal exposure, ensuring that participants could acquire both associations and were equally familiar with both associations at the time of the second expectancy violation. This design controlled for differences in learning history that might otherwise impact performance estimates and more clearly dissociated effects of maintained priors from conventional intertrial priming experiments based on intermixing colors unforeseeably across trials. It also allowed us to test whether attention guidance to the target benefits or suffers when reinstating a previously learned target-shape–color association.

If swapping back to a previously conclusively learned target-shape–color association eliminates or increases costs during the second expectancy violation (i.e., during swapping back to Prior A), we expected an interaction between the variables phase in the experiment or interval (of three trials at the end of learning of a prior/precritical phase; during the immediately following change to an alternative target-shape–color association/critical phase; and immediately following the change/postcritical phase) and order of the expectancy violation (first, second), reflected in search-time costs emerging either only during the first but not during the second expectancy violation or being stronger at the second than at the first expectancy violation, respectively. The first pattern would indicate that participants can establish, maintain, and flexibly reactivate two different learned priors within the same experimental context. The second pattern would be in line with the establishment and maintenance of two priors—one per each of two learned target-shape–color associations too—but would suggest that participants represent the two priors as mutually exclusive alternatives. In contrast, persisting swapping costs of about equal amounts across both expectancy violations would suggest limitations in maintaining or reusing two distinct priors, consistent with an updating of a single prior.

Finally, in Experiment 2, we varied target salience to examine whether this modulated (back-)swapping costs to the first established prior during a second expectancy violation (relative to forward-swapping costs to a second prior during the first expectancy violation). Even if Experiment 1 yields little or no evidence for differences in swapping cost between the first and second expectancy violation, increased salience may strengthen the impact of learned priors via its interaction with the guidance of attention to the targets [26], thereby facilitating participants’ establishment of a corresponding prior for each of two stimulus-color assignments.

## 2. Experiment 1

### 2.1. Materials and Methods

#### 2.1.1. Participants

Forty participants (32 female, mean age: 22.4 years, range 19–36 years) took part on a voluntary basis. All procedures were conducted in accordance with the Declaration of Helsinki and relevant Austrian ethical standards, and all participants (of all three experiments) provided written informed consent prior to participation.

#### 2.1.2. Apparatus

Eye movements were recorded using the EyeLink 1000 (SR Research Ltd., Mississauga, ON, Canada) at 1000 Hz. Head position was stabilized using a chin rest with a forehead strip, and the proper signal was controlled prior to each trial and recalibrated if necessary. Stimulus materials were presented on a 19-inch CRT monitor with a refresh rate of 120 Hz and a resolution of 1024 × 768 pixels at a viewing distance of 57 cm. Stimulus presentation was controlled using Experiment Builder (SR Research, version 1.6).

#### 2.1.3. Stimuli and Procedure

As stimulus, predefined target letters, here, the letters U or H, were used and presented alongside distractor letters (A, B, C, D, E, F, L, P and S). Letters were typed in the font Times New Roman and had a height of 1° of visual angle. They were displayed above and below screen center, at a distance of 2° of visual angle from the center of the monitor. Letters were either red or green (both 65 cd/m^2^) on a neutral grey background (80 cd/m^2^). The order and combination of target and distractor letters were randomized. The response keys for the target position (i.e., keys ‘x’ and ‘comma’, to be pressed with the left and right index finger, respectively) were consistently assigned to the positions of the target (at the top vs. at the bottom), and S-R mappings were balanced across participants. Initial (and back-swapped) versus swapped stimulus-color assignments (or target and distractor colors, i.e., green vs. red, or vice versa) were also balanced across participants.

The session started with eight practice trials before the data collection began. Each trial started with a fixation cross (duration between 1 and 5 s) at the screen center, followed by the simultaneous presentation of a target and a distractor letter, one above and one below the center of the monitor, respectively (with positions randomly varying across trials). This target display remained on until a button press or until a time-out after 1500 ms.

**Figure 1:**
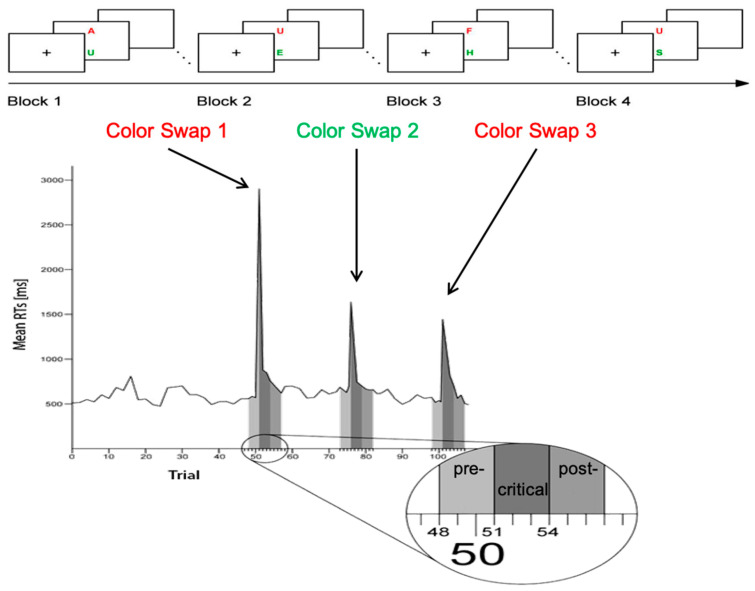
General procedure and corresponding analysis. In this example of sequences of trials (upper row), the first expectancy violation or color swap appeared after 50 trials (hence ‘Group 50’ for these participants), whereas another group of participants (‘Group 75’) was presented with two color swaps between three blocks in total only. In the lower row, one can see that performance (here, search reaction times; RTs) for different phases (precritical, critical, postcritical) was averaged across three consecutive trials each, nine consecutive trials altogether.

Participants were instructed to fixate the target with their eyes and to report the position of the target letter (with a predefined button). Critically, for the first 50 (or, between participants, for the first 75) trials, and then from Trial 51 to 100 (or, between participants, from Trial 76 to 150), and again from Trial 101 to 150 (only in the group of participants with a first precritical period of 50 color-similar trials), a fixed color (i.e., red or green) was assigned to the target and distractor letters, respectively. This was done in order to allow sufficient learning per each target-shape–color association and foster the participants’ expectancy of a specific (target) color over these trials. At the outset, over the first 50 trials (i.e., in the precritical phase or interval), participants could notice that the target letter was always presented in the same color and use this color information to recognize targets faster. After 50 or 75 trials (between participants) an unexpected color swap of target and distractor colors occurred. This was called the critical or color-swap trial (but measurements of the effects of the expectancy violation were taken as averages across the first three trials of a swap, see below). This was followed by an adaptation to this change (the postcritical phase). In terms of search reaction times (RTs), we expected an increase for the critical trials compared to the precritical phase and a subsequent decrease in RTs in the postcritical trials (see Figure 1). In addition, based on past findings [12], we expected a difference in the guidance of attention to the targets versus distractors in the critical trials because both stimuli provided critical prediction-error information.

#### 2.1.4. Analysis

Search reaction times (RTs) and eye movement data were recorded. For further analyses, the between-participants variable group (Group 50 vs. Group 75) was established, depending on the number of trials, after which the first and the second unexpected color swap occurred. Both color swaps were not announced. Data derived from three consecutive trials directly before (precritical), during (critical), and after the color swap (postcritical) were used as steps of the independent variable interval. The independent variable order indicated the temporal position of the color swaps (first, second, and—for Group 50 only—third swap).

Averaging across three trials per condition was done for three interconnected reasons. First, a period of three trials was chosen to fully represent the effect of the expectancy violation, since past data indicated that the impact of an expectancy violation can last longer than just a single trial [9]. In the present experiment, this was even more necessary because feedback about the correctness of the current trial’s response was not provided and one error was allowed for data to be retained, meaning that participants might have noticed the expectancy violation sometimes not in the critical trial itself but in one of the two trials following the critical trial. Second, for the precritical and postcritical trials, we averaged only across three consecutive trials, too, to roughly equate the averages for the signal-to-noise ratio of the performance estimate (i.e., it was based on three trials for each phase). Third, we used nine consecutive trials to account for general learning effects with the task at hand across the three different phases or intervals (the three last precritical phase trials; Trials 1 to 3 following the swap as the critical phase; and Trials 4 to 6 following the swap as the postcritical phase sample). Note that the blocks of the experiment were noticeable for the participants only for the swapped colors of targets versus distractors. Note on the colors in Figure 1: If the color version of Figure 1 is not available to you, the reader, please note that the target versus distractor was consistently colored across trials, except for the critical trials, in which these colors were swapped.

### 2.2. Results

#### 2.2.1. Search Reaction Time Analysis

Repeated-measurement analyses of variance (ANOVAs) were conducted, and in case of violation of sphericity, the Greenhouse–Geisser correction was applied. Post hoc tests were Bonferroni corrected. For further analysis, all participants making more than one error (in the further investigated trials) were excluded from further analysis, leading to a sample size of *N* = 29. Furthermore, all search reaction times (RTs) deviating more than two *SD*s from the mean (calculated for each combination of independent variable and participant separately) were also excluded.

**Figure 2:**
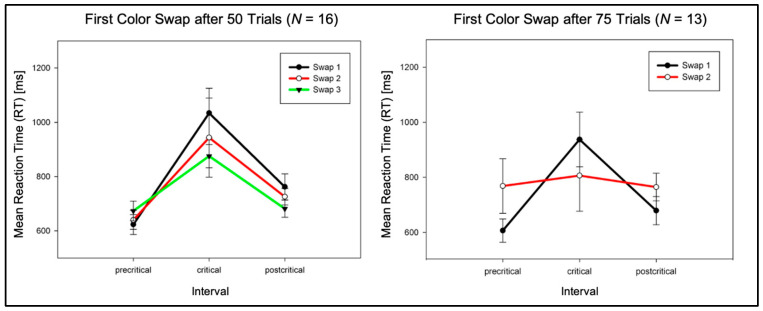
Mean search reaction times (RTs; error bars indicate *SE*s of the means) on the *y* axis as a function of interval (precritical, critical, postcritical) on the *x* axis and of swap position (Swap 1: black line/filled circles, Swap 2: red line/white circles, Swap 3, only in Group 50: green line/black triangles) of Experiment 1. On the left, results of Group 50. On the right, results of Group 75.

We first investigated differences between the three color swaps for Group 50 (*N* = 16; see Figure 2, left panel). A 3 × 3 repeated-measurements ANOVA, with the independent variables interval (precritical vs. critical vs. postcritical) and order (first vs. second vs. third swap), on mean search RTs led to the following results: A significant main effect for the variable interval was found, *F*(1.3, 20.0) = 20.02, *p* < 0.001, η_p_^2^ = 0.57. Post hoc Bonferroni-adjusted *t*-tests confirmed significant differences between all three phases: precritical (646 ms), critical (950 ms), and postcritical trials (723 ms, all *p*s < 0.05), averaged over all three swaps. No further significant effects were found in this analysis (all *p*s > 0.48, all *F*s < 0.44).

The next analysis compared the two groups (see Figure 2) and was limited to the first two color swaps (*N* = 29), as the third color swap was not realized in Group 75. A 3 × 2 × 2 repeated-measurements ANOVA, with the independent variables interval (precritical vs. critical vs. postcritical), order (first vs. second swap), and the between-participants variable group (Group 50 vs. Group 75), on mean search RTs, led to a significant main effect for interval, *F(*1.6, 43.5) = 13.4, *p* < 0.001, η_p_*^2^* = 0.33. Post hoc Bonferroni-adjusted *t*-tests confirmed significantly higher search RTs in critical (930 ms) compared to precritical (660 ms) and postcritical trials (733 ms; both *p*s < 0.01), whereas pre- and postcritical trials showed no significant difference (*p* = 0.36).

#### 2.2.2. Eye Movement Analysis

**Figure 3:**
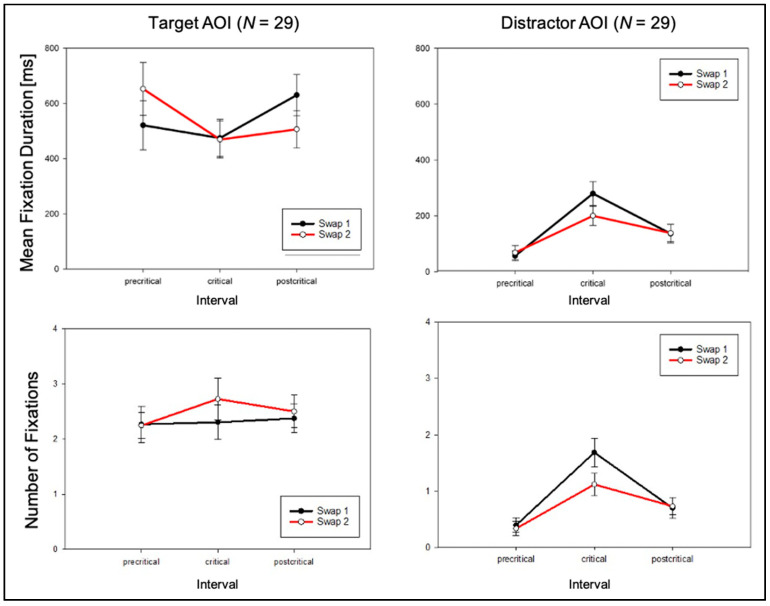
Mean fixation durations (in ms; **upper** panel) and numbers of fixations (**bottom** panel) on the *y* axis as a function of interval (precritical, critical, postcritical) on the *x* axis and of the order position of the critical trial (Swap 1: black line/filled circle vs. Swap 2: red line/white circle) for the target area of interest (AOI) (**left** panels) and distractor AOI (**right** panels) in Experiment 1. Error bars indicate *SE*s of the respective means.

The same exclusion criteria as in the search RT analysis were applied here (*N* = 29). For the analysis of eye movements toward the target or distractor letter, corresponding areas of interest (AOIs) were defined. Both AOIs were circles centered on the target or distractor letter and covered 2° of visual angle (=56 pxs diameter; letter height was 1° of visual angle = 28 pxs). Mean fixation durations (see Figure 3) as well as number of fixations in the two AOIs were compared.

##### Mean Fixation Duration

A 3 × 2 × 2 × 2 repeated-measurements ANOVA, with the independent variables interval (precritical vs. critical vs. postcritical), order (first vs. second swap), AOI (target vs. distractor), and the between-participants variable group (Group 50 vs. Group 75), on mean fixation durations, led to the following results: We found a significant main effect for AOI, *F*(1, 27) = 89.4, *p* < 0.001, η_p_*^2^* = 0.77, with generally longer fixation durations on the target (542 ms) compared to the distractor letter (146 ms). A significant interaction between interval and AOI was found, *F*(2, 54) = 6.3, *p* < 0.01, η_p_^2^ = 0.19. Post hoc Bonferroni-adjusted *t*-tests confirmed significant differences between all intervals for the AOI distractor letter (all *p*s < 0.05), but not for the target letter AOI (all *p*s > 0.38).

##### Number of Fixations

A 3 × 2 × 2 × 2 repeated-measurements ANOVA, with the independent variables interval (precritical vs. critical vs. postcritical), order (first vs. second swap), AOI (target vs. distractor), and the between-participants variable group (Group 50 vs. Group 75), on the number of fixations, led to the following results: A significant main effect for interval was found, *F*(2, 54) = 7.8, *p* < 0.01, η_p_^2^ = 0.22. Post hoc Bonferroni-adjusted *t*-tests confirmed a significant difference only between precritical (1.3) and critical trials (2.0, *p* < 0.01; all other *p*s > 0.10). A significant effect for AOI was obtained, *F*(1, 27) = 71.0, *p* < 0.001, η_p_^2^ = 0.73, too, with generally more fixations on the target (2.4) compared to the distractor letter (0.8), which reflected the general instruction to fixate on the target letter. A significant interaction between interval and AOI was found, *F*(2, 54) = 3.4, *p* < 0.05, η_p_^2^ = 0.11. Post hoc Bonferroni-adjusted *t*-tests confirmed significantly more fixations on the distractor in critical (1.4) compared to pre- or postcritical trials (0.4 and 0.7, both *p*s < 0.01), but no significant differences between all intervals for the target letter AOI (all *p*s > 0.99).

### 2.3. Discussion

Findings in Experiment 1 showed that expectancy violations reliably increased search reaction times, whereas evidence for modulation of these costs by swap order or reinstated target-shape–color associations was limited. Neither search RTs nor eye movements indicated interactions between interval and order of color swaps, suggesting that experience with a prior association did not eliminate or reliably modulate the impact of subsequent expectancy violations.

In line with previous findings, eye movement data showed more fixations on the distractor in the critical, color-swap trials, and this effect numerically decreased across subsequent color swaps (see Figure 3, right). However, as for the search RTs, no significant interactions between interval and order were obtained in this context. Distractors were generally fixated more often and on average longer in critical trials than in pre- or postcritical trials, independently of the order of the color swap or group. Together, these findings indicated that previously learned target-shape–color associations did not attenuate or increase attentional guidance during expectancy violations when swapping back to a learned association. However, the role of previously learned target-shape–color associations during repeated swapping remained inconclusive across experiments.

In Experiment 2, we tested whether stimulus salience, manipulated via set size, influences the impact of prior knowledge during both forward-swapping and back-swapping conditions.

## 3. Experiment 2

In Experiment 2, we tested a possible effect of salience on the impact of prior knowledge with the target-shape–color associations of the two violations of the expectancy—that is, during (forward) swapping and swapping back. To that end, we varied the number of distractor letters that were presented together with the target (one, five, or 11). These were our hypotheses: If salience provided a bottom-up source for the guidance of attention to the singleton target, this could further mitigate any possible difference between the first expectancy violation (forward-swapping) and the second expectancy violation (back-swapping). However, if salience (also) supported participants’ successful establishment of the different specific priors (or representations of target shape-color assignments) in the first place, an interaction reflecting stronger forward-swapping costs during the first expectancy violation than back-swapping costs during the second expectancy violation might be observed, as long as a higher salience supports the registration or encoding of the currently applying target-shape–color association in at least a subset of all trials. The reader should note that salience was only manipulated before and after the critical trials. In contrast, the critical trials were all low set-size conditions, with a single target and a single distractor. This was done for the sake of better comparability of findings across Experiments 1 and 2. Note that this does not mitigate any helpful role of increased salience for participants’ learning of the color–shape associations before or after the critical trials.

Experiment 2 also allowed us to investigate if participants used a color search strategy at all. To note, participants could have always searched for the two shapes of the target letters to find the target. At least in the critical trials, this was not unlikely once participants noticed that a search template for the last associated target color directed attention to the distractor. However, in the other trials, especially following a color swap, this was less clear. Following a swap, participants could have abstained from a color-search strategy at all, simply because this was not a successful strategy in the critical trials. Specifically, participants could have strategically dropped the usage of accessory color information about targets following the first color swap (see Section 1). In Experiment 1, this was not reflected in a lack of or reduction in back-swapping costs. However, a bottom-up priming effect of trial-by-trial target color repetition, so it existed, could have worked even during shape search. Thus, the back-swapping costs are not entirely conclusive regarding the participants’ search strategy. Luckily, a set-size manipulation in the trials following the swap (i.e., following the critical trials) and the comparison of the set-size effect before and following the swap provide an independent measure of this possibility. If participants used letter search, we expected that search times would increase with the number of stimuli in the display (i.e., with set size). However, a color-search strategy should lead to a relatively flat search function that does not increase with set size [27]. Based on the consistent swapping costs observed across consecutive expectancy violations in Experiment 1 that, we believe, do not represent mere “bottom-up color priming” but rather reflected participants’ persistent usage of shape-associated colors to search for targets even following a swap, we thought it was more likely that participants used the learned and, thus, expected target colors to search for the targets. This would be reflected in a lack of a set-size effect in both precritical and postcritical trials (i.e., before and after a swap or an expectancy violation).

### 3.1. Materials and Methods

#### 3.1.1. Participants

Twenty-five participants (26 female, mean age: 24.9 years, range 19–64 years) took part on a voluntary basis.

#### 3.1.2. Stimuli and Procedure

**Figure 4:**
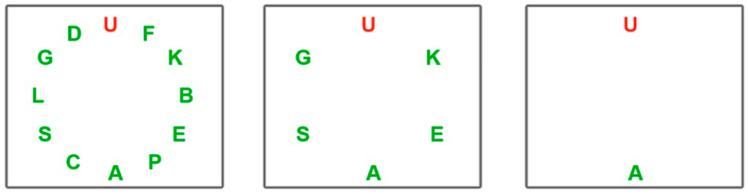
Possible set sizes in Experiment 2, with 12, six, or two letters that were displayed in the target display in high (**left**), medium (**center**), and low set-size/salience conditions, respectively. As before, the task was to indicate the position of a predefined target letter (U or H).

The same procedure was used as described in Experiment 1. Stimuli were the same target and distractor letters, but either two, six, or 12 letters were presented per target display (see Figure 4). Letters were arranged in a circle, at a distance of 2° of visual angle from the center of the screen. Trials were presented in a randomized order, but, in all three critical trials (i.e., all three trials following an expectancy violation), only one distractor was shown alongside the target (comparable to the same conditions in Experiment 1). This was done for better comparison to the critical trials in Experiment 1.

### 3.2. Results

For our tests, participants with three incorrect responses after the critical trial were removed from further analysis because with three incorrect responses, it is safe to assume that participants did either not notice the color swap in the critical trial(s) or did not follow the instructions. Due to the application of this criterion, our analyzed (final) sample size in the following reports is *N* = 14.

#### 3.2.1. Search Reaction Time Analysis

**Figure 5:**
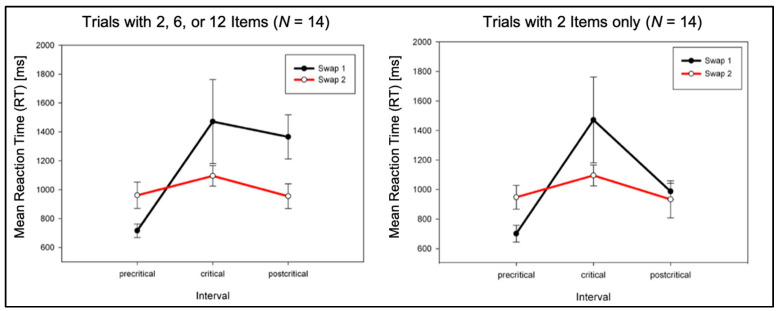
Mean search reaction times (RTs; error bars indicate SEMs) on the *y* axis as a function of interval (precritical, critical, postcritical) on the *x* axis and of order of swap (Swap 1: black line/filled circle, Swap 2: red line/empty circle) in Experiment 2.

A 3 × 2 × 2 repeated-measurements ANOVA, with the independent variables interval (precritical, critical, postcritical) and order (first swap, second swap), and the between-participants variable group (Group 50 vs. Group 75), on mean search RTs, led to a significant main effect for interval, *F*(1.3, 15.7) = 9.0, *p* < 0.01, η_p_^2^ = 0.43. Participants showed significantly longer search RTs in critical (1283 ms) compared to precritical trials (838 ms; *p* < 0.05) but not in comparison to postcritical trials (1160 ms; *p* = 0.92). Furthermore, pre- and postcritical trials also significantly differed from one another (*p* < 0.01). In addition, a significant interaction between interval and order was found, *F*(1.5, 18.2) = 4.8, *p* < 0.05, η_p_^2^ = 0.29. Post hoc *t*-tests confirmed significant differences for the first color swap between precritical (715 ms) and postcritical trials (1365 ms, *p* < 0.01), but (barely) non-significant differences for critical trials (1471 ms, *p* = 0.051) and no significant differences between the different intervals of the second target-shape–color association (back-)swaps (all *p*s > 0.19). No further significant effects were found in this analysis (all *p*s > 0.10, all *F*s < 3.1). See Figure 5 for the results.

#### 3.2.2. Analysis Concerning Participants’ Search Strategy: Letters or Colors?

To test what search strategy was used before versus after the first critical trial (or forward swap to a novel, hitherto not experienced target-shape–color association), data was first divided into two time brackets: before (i.e., initial phase) and after the first expectancy-violating color swap (i.e., later trials). All nine trials falling in the precritical, critical, or postcritical phase of the first color swap were excluded. (Furthermore, all three trials in the precritical phase of the second swap and later were not analyzed.) Depending on the number of items in the display, set size was defined as low (two items), medium (six), or high (12).

**Figure 6:**
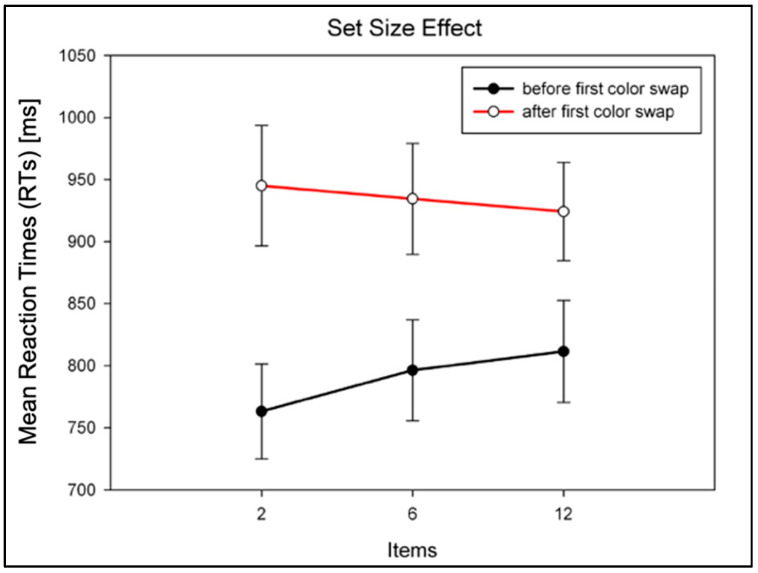
Mean search reaction times (RTs; error bars indicate SEMs) on the *y* axis as a function of the set size (two, six, or 12 items) on the *x* axis and of the time bracket (before vs. after the first color swap) in Experiment 2.

A 2 × 2 repeated-measurements ANOVA, with the independent variables time bracket (before vs. after the first swap), set size (low vs. medium vs. high), and the between-participants variable group (Group 50 vs. Group 75), on mean search RTs, led to the following results: A significant main effect for the variable time bracket was found, *F*(1, 12) = 13.7, *p* < 0.01, η_p_^2^ = 0.53. Post hoc *t*-tests confirmed significantly shorter search RTs before (769 ms) than after the first color swap (931 ms). No further significant effects were found in this analysis (all *p*s > 0.25, all *F*s < 1.0). As can be seen in Figure 6, set size had no significant effect. This pattern is broadly consistent with the possibility that participants relied on color-guided search.

#### 3.2.3. Eye Movement Analysis

Exclusion criteria and analysis described in Experiment 1 were also applied here.

##### Mean Fixation Duration

**Figure 7:**
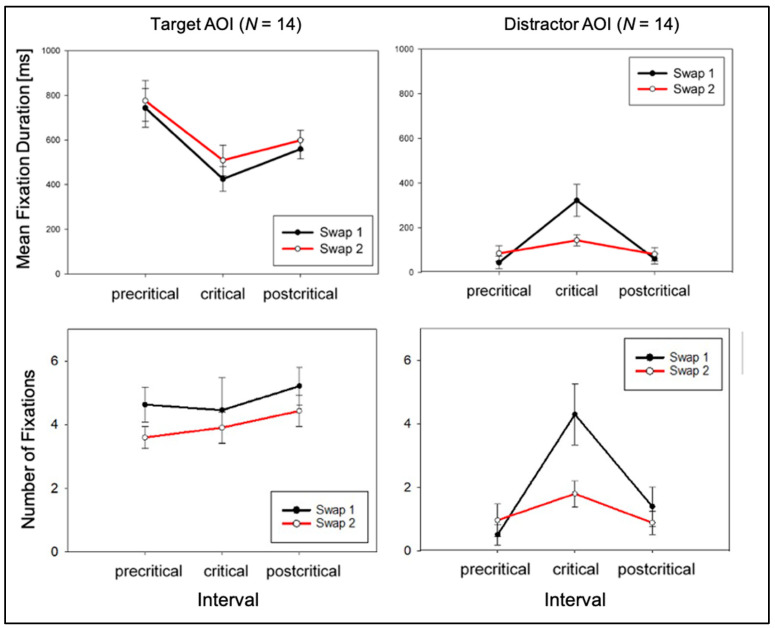
Mean fixation durations (in ms; **top** panel) and numbers of fixations (**bottom** panel) on the *y* axis as a function of interval (precritical, critical, postcritical) on the *x* axis and of order position (Swap 1: black line/filled circles, Swap 2: red line/empty circles) in Experiment 2. Results for the area of interest (AOI) of the target are on the left, and results for the AOI of the distractor are on the right. Error bars indicate SEs of the respective means.

A 3 × 2 × 2 × 2 repeated-measurements ANOVA, with the independent variables interval (precritical, critical, postcritical), order (first swap, second swap), AOI (target, distractor), and the between-participants variable group (Group 50, Group 75), on mean fixation durations, led to the following results: We found a significant main effect for AOI, *F*(1, 12) = 91.9, *p* < 0.001, η_p_^2^ = 0.88, with generally longer fixation durations on the target (602 ms) compared to the distractor letter (123 ms). A significant interaction between order and AOI was found, *F*(1, 12) = 6.7, *p* < 0.05, η_p_^2^ = 0.36. Post hoc *t*-tests confirmed significantly longer fixation durations on the target compared to the distractor letter for both color swaps (both *p*s < 0.001). A significant interaction between interval and AOI, *F(*1.3, 15.1) = 11.9, *p* < 0.01, *η_p_^2^* = 0.50, was further investigated by post hoc *t*-tests that confirmed significant differences between precritical and critical trials for the AOI distractor letter (*p* < 0.01), as well as between critical and postcritical trials for the target letter AOI (*p* < 0.05). No further significant effects were found in this analysis (all *p*s > 0.52, all *F*s < 3.5). See also Figure 7.

##### Number of Fixations

A 3 × 2 × 2 × 2 repeated-measurements ANOVA, with the independent variables interval (precritical, critical, postcritical), order (first swap, second swap), AOI (target, distractor), and the between-participants variable group (Group 50, Group 75), on number of fixations, led to the following results: A significant main effect for order was found, *F*(1, 12) = 5.2, *p* < 0.05, η_p_^2^ = 0.30, with generally more fixations in the first (3.4) compared to the second color swap (2.6). A significant main effect for interval was found, *F*(1.7, 20.6) = 5.1, *p* < 0.05, η_p_^2^ = 0.30. Post hoc *t*-tests confirmed a significant difference only between precritical (2.4) and critical trials (3.6, *p* < 0.05; all other *p*s > 0.23). A significant effect for AOI, *F*(1, 12) = 54.5, *p* < 0.001, η_p_^2^ = 0.82, reflected generally more fixations on the target (4.4) compared to the distractor letter (1.6). A significant interaction between interval and AOI was found, *F*(1.8, 21.3) = 12.8, *p* < 0.001, η_p_^2^ = 0.52. Post hoc *t*-tests confirmed that the distractor letter was more often fixated in critical compared to pre- and postcritical trials (both *p*s > 0.01), but no significant differences for the target letter AOI were found (all *p*s > 0.34). A significant interaction between order, interval, and AOI was also found, *F*(1.6, 19.0) = 5.2, *p* < 0.05, η_p_^2^ = 0.30. Post hoc *t*-tests confirmed significantly more fixations on the target compared to the distractor letter in all intervals for the color swaps (all *p*s > 0.01), with only one exception: No significant difference between the AOIs was found in the critical trials of the first color swap (*p* = 0.86). This finding indicates that participants in general followed the instruction to fixate on the target, but the distractor attracted attention in the first expectancy-violating trial so as to abolish the otherwise generally more likely fixations on the target. No further significant effects were found in this analysis (all *p*s > 0.06, all *F*s < 3.3). For the results, see Figure 7.

### 3.3. Discussion

In Experiment 2, we implemented a set-size variation in order to gain further insights into our previous results on attention guidance and visual-search delays through expectancy violations. Search reaction time data indicated again a cost for the first expectancy violation—a (forward-) swap of the target-shape–color association to a novel association. However, again, no significant difference between the first and second expectancy violations was observed, despite using a salience manipulation that could have increased learning and, thus, the impact of foreknowledge of the already experienced target-shape–color associations during the second expectancy violation. The only evidence that indicated a stronger impact of the first than of the second expectancy violation was seen in the probability of fixations on the target versus the distractor. In line with a mitigating influence of foreknowledge on the second expectancy violation, the probability of fixations on targets versus distractors was the same during forward swapping, but, as instructed, more fixations on targets versus distractors were found during the second expectancy violation. Accordingly, the swap-order effect observed in Experiment 1 was not consistently replicated in Experiment 2. This finding would be in line with a lower cost of the second expectancy violation and with an origin of the costs of the first expectancy violation in terms of (erroneous) attention guidance (to the distractors). However, because this effect was neither found in the manual search reaction times nor in the fixation durations, it needs to be interpreted with caution and constitutes spurious evidence for a difference in swapping costs between the first and second expectancy violations at best.

In addition, no significant influence of set size was found either. If anything, manual search RTs showed a tendency to increase with increasing set size before the first expectancy violation, but definitely not after the first expectancy violation. These results speak for a color-search strategy because we would have expected a set-size effect—that is, increasing search times with an increasing number of stimuli per each target display if our participants would have searched for the shapes to find the target letters.

## 4. General Discussion

In the current study, we investigated the influence of prior knowledge (or priors, short for representations of prior probabilities, here about target-shape–color association) on attention guidance during expectancy violations. To that end, our participants had to visually search for target letters (and report their positions). Critically, target versus distractor letters were consistently colored (e.g., in red vs. green), but during a first and a second expectancy violation, we presented our participants with swapped stimulus-color assignments (e.g., if the target was red and the distractor was green in the precritical trials, before the swap, the target was green and the distractor was red in the critical and postcritical trials, following the swap). Importantly, the first expectancy violation concerned a single (experimentally induced) prior, whereas the second expectancy violation consisted of swapping back to the original target-shape–color association and, hence, a return to a potentially pre-existing prior. Because priors should facilitate perception, we tested whether swapping costs could be abolished or would even increase during the second expectancy violation. Both possibilities would be in line with the maintenance of two priors—one for the first target-shape–color association and one for the second target-shape–color association—either as two independent options to use for attention guidance or as mutually exclusive alternatives.

However, support for these possibilities was limited and not consistently observed across experiments. Across two experiments, we found some evidence for quantitative but not significantly smaller costs for the second versus first expectancy violation. It might be argued that this was due to bottom-up priming of target colors that was present even during the search for target letters. Specifically, following the first color swap, participants might have switched to shape search to find the targets because they no longer trusted a target-color–shape association. However, in Experiment 2, even following a color swap, search time was not significantly modulated by set size, a finding much better in line with color-based than shape-based searches. Likewise, though longer manual search RTs during expectancy violations (in the critical trials, “during” the swap, than in the pre- and postcritical trials, before and after the swap, respectively) went hand in hand with altered attention guidance (e.g., more evidence for eye movements to the distractors in critical than pre- and postcritical trials), eye movement measures were consistent with persistent swapping costs during the second expectancy violation. All in all, these results are more consistent with the updating of a single currently dominant prior than with the stable maintenance of two fully independent priors of target-shape–color associations.

Importantly, these findings can be interpreted within accounts of prediction-error-driven learning and adaptive attentional control. From this perspective, expectancy violations induce updating because the current prediction about target-defining features becomes unreliable. Rather than maintaining multiple fully independent priors in parallel, the system may rely on the most recently successful representation to guide attention in changing environments alone. Accordingly, repeated expectancy violations continue to produce measurable processing costs because prediction errors necessitate updating and reconfiguration of attentional guidance. This interpretation is consistent with accounts in which behavior is shaped by the statistical structure of the environment, where recent information is weighted more strongly than older information, and forgetting can support adaptive inference [28,29].

The back-swapping effect was found despite a substantial experience with each of the two target-shape–color associations for an extended period of consistent target-shape–color associations of at least 50 trials. Thus, the present results in favor of the updating of a single prior cannot be explained by the fact that intermixing of different target colors encouraged short-time updates of a single prior at the expense of establishing a second prior. Instead, the findings are more consistent with adaptive prioritization of the most recently successful representation, even after extensive learning of alternative associations. This interpretation is also consistent with accounts emphasizing that behavior is shaped by environmental structure and recency-weighted sampling of experience [28,29]. Our findings are also consistent with interpretations in terms of bottom-up priming of attention capture [8], although this interpretation is less likely, as intertrial priming effects have been shown to be susceptible to contextual and top-down influences (e.g., [30,31,32]). For example, Fecteau (2007) found intertrial priming effects only for the searched-for task-relevant features of the target [31]. In fact, based on the current study, it is more likely that seeming evidence of bottom-up priming actually reflected the updating of a single target-directed prior for those features that guided attention towards the target.

In line with this interpretation, participants’ search behavior for target letters indicated continued reliance on target color rather than target shape or identity information. In Experiment 2, we found that a variation in set size (i.e., the number of concurrent stimuli per search display) had no significant effect on search times. This pattern is broadly consistent with color-based as opposed to letter-based search, the latter of which would predict increasing search times with increasing set sizes (cf. [27]). Similar results have been reported previously, with Weichselbaum et al. [33] (Experiment 1) also providing evidence for a color-based search strategy (in the form of more attention capture by similar target-color distractors than dissimilar ones), likely reflecting better efficiency of color- than shape-based search.

Limitations of the present study include that only a relatively small number of trials could be included in the analyses, reflecting the strict experimental design required to isolate the two expectancy violations and swapping conditions. In addition, the final sample sizes following exclusions were relatively small, which may have reduced statistical power, particularly for detecting subtle effects. This may have reduced sensitivity to detect subtle differences between forward- and back-swapping costs. In addition, the design does not allow strong conclusions about the underlying mechanisms of bottom-up priming versus prior-based guidance, which cannot be disentangled. Another limitation concerns the artificial character of our laboratory experiment. It might be that participants’ general expectation of little systematic relationship between target features during laboratory experiments lured them to update a single prior instead of maintaining two priors, and that the maintenance of two priors is more typical of real-world situations in which meaningful differences between situations require discrimination learning—that is the acquisition and maintenance of more than one prior. For instance, humans may sensibly maintain one prior for the searching of red raspberries from June to October in addition to another prior for the searching of black currants between mid-June and August. In addition, although salience was manipulated in Experiment 2, it did not modulate swapping costs, leaving open its role under these conditions. Finally, we did not theoretically reflect upon or empirically dissociate guidance towards target features and suppression of distractor features. To note, distractor-feature assignments were also consistent and swapped across phases, meaning that in theory all swapping costs could be due to target search and distractor suppression. We do not necessarily consider this last limitation as a major drawback because, in fact, participants could have established priors for targets and distractors. This should have increased the sensitivity of our design for the maintenance of one prior versus two priors.

## 5. Conclusions

With prior knowledge of the relationships between visual features, one might hope not to fall for a repeated expectancy violation twice. However, from the perspective of learning theories each expectancy violation provides a learning opportunity in the form of a prediction error for the correction or updating of a currently used or dominant representation of prior probabilities (e.g., of relationships between different visual features). In line with the adaptive value of such prediction errors, we found that humans reliably showed costs following repeated expectancy violations, even after substantial prior experience with the relevant target-color associations. However, the extent to which previously learned associations modulate later expectancy violations remains to be clarified by future research.

## Data Availability

The data that support the findings of this study are available from the corresponding author upon reasonable request in order to protect the privacy of the participants.
